# 1-[2,2-Bis(1,3-benzimidazol-1-ylmeth­yl)-3-bromo­prop­yl]-1,3-benzimidazole

**DOI:** 10.1107/S1600536811023464

**Published:** 2011-06-30

**Authors:** Tai-Bao Wei, Yan-Yun Lu, Cheng Cao, Hong Yao, You-Ming Zhang

**Affiliations:** a College of Chemistry and Chemical Engineering, Key Laboratory of Eco-Environment-Related Polymer Materials of the Ministry of Education, Gansu Key Laboratory of Polymer Materials, Northwest Normal University, Lanzhou 730070, People’s Republic of China

## Abstract

The title compound, C_26_H_23_BrN_6_, has been synthesized as a potential ligand for the construction of metal–organic frameworks. The three benzimidazolyl groups present three potential coordination nodes. The dihedral angles between the benzimidazole ring systems are 74.03 (10), 66.49 (9) and 74.09 (9)°. The structure contains large voids, which contain highly disordered solvent mol­ecules that may be CH_3_CH_2_OH. Since the solvent mol­ecules could not be located, the *PLATON*/*SQUEEZE* procedure [Spek (2009 ▶). *Acta Cryst.* D**65**, 148–155] was used.

## Related literature

For applications of metal organic frameworks, see: Ferey *et al.* (2005 ▶); Bradshaw *et al.* (2005 ▶); Pan *et al.* (2004 ▶); Ko *et al.* (2002 ▶); Pan *et al.* (2006 ▶); Barnett & Champness (2003 ▶); Yang *et al.* (2003 ▶); Liu *et al.* (2010 ▶). For a related structure, see: Clegg & Martin (2007 ▶).
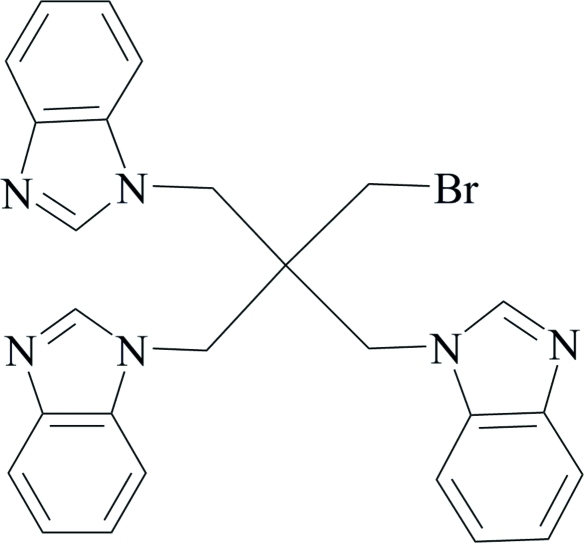


## Experimental

### 

#### Crystal data

C_26_H_23_BrN_6_
*M* *_r_* = 499.41
Triclinic, 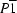

*a* = 9.297 (4) Å
*b* = 11.869 (5) Å
*c* = 13.661 (6) Å
α = 68.956 (4)°
β = 77.398 (4)°
γ = 84.805 (4)°
*V* = 1372.9 (10) Å^3^
*Z* = 2
Mo *K*α radiation
μ = 1.52 mm^−1^
*T* = 293 K
0.32 × 0.30 × 0.29 mm

#### Data collection

Bruker APEXII CCD diffractometer
Absorption correction: multi-scan (*SADABS*; Sheldrick, 2008*a* ▶) *T*_min_ = 0.642, *T*_max_ = 0.667
9233 measured reflections
5012 independent reflections
3417 reflections with *I* > 2σ(*I*)
*R*_int_ = 0.025

#### Refinement

*R*[*F* ^2^ > 2σ(*F* ^2^)] = 0.037
*wR*(*F* ^2^) = 0.086
*S* = 1.01
5012 reflections
299 parameters
H-atom parameters constrained
Δρ_max_ = 0.41 e Å^−3^
Δρ_min_ = −0.20 e Å^−3^

### 

Data collection: *APEX2* (Bruker, 2008 ▶); cell refinement: *SAINT* (Bruker, 2008 ▶); data reduction: *SAINT*; program(s) used to solve structure: *SHELXS97* (Sheldrick, 2008*b* ▶); program(s) used to refine structure: *SHELXL97* (Sheldrick, 2008*b* ▶); molecular graphics: *SHELXTL* (Sheldrick, 2008*b* ▶); software used to prepare material for publication: *SHELXTL*.

## Supplementary Material

Crystal structure: contains datablock(s) I, global. DOI: 10.1107/S1600536811023464/fy2012sup1.cif

Structure factors: contains datablock(s) I. DOI: 10.1107/S1600536811023464/fy2012Isup2.hkl

Supplementary material file. DOI: 10.1107/S1600536811023464/fy2012Isup3.cml

Additional supplementary materials:  crystallographic information; 3D view; checkCIF report

## Figures and Tables

**Fig. 1.**
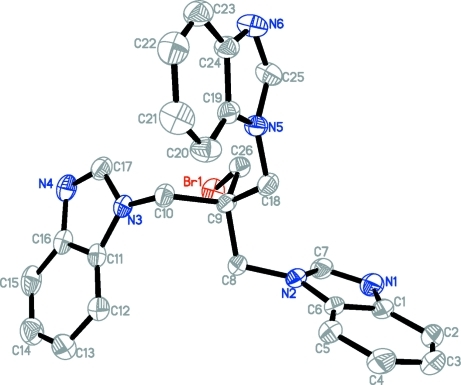
The structure of the title compound, showing the atom labelling scheme. Displacement ellipsoids are drawn at the 30% probability level. H atoms have been omitted for clarity.

## supplementary crystallographic information

### Comment

In recent years, metal-organic frameworks (MOFs) with network structures have received remarkable attention as a unique class of multifunctional hybrid materials with potential applications in such fields as sorption, molecular separation, luminescence, catalysis, and magnetism (Ferey *et al.*, 2005; Bradshaw *et al.*, 2005; Pan *et al.*, 2004; Ko *et al.*, 2002; Pan *et al.*, 2006). Several N-donor bridging ligands have been investigated for the construction of novel MOFs, for example 1,3-bis(4'-pyridyl)propane, 1,4-bis(4-pyridyl)butane (Barnett *et al.*, 2003), bis(2-benzimidazolylmethyl)(2-pyridylmethyl)amine and bis(2-pyridylmethyl)(2-benzimidazolylmethyl)amine (Yang *et al.*, 2003), 1,1' -(1,4-butanediyl)-bis[2-(2-pyridyl)benzimidazole], 1,1'-(1,6-hexanediyl)bis[2-(2-pyridyl)benzimidazole] and 1,1'-(1,1'-decanediyl)bis-[2-(2-pyridyl)benzimidazole] (Liu *et al.*, 2010).

We designed and synthesized the title compound to be applied as a tridentate ligand. The molecular structure is show in Fig. 1. This ligand is expected to be a good candidate for the construction of coordination polymers with diverse structures. First, the three benzimidazole N atoms of the ligand can act as three potential coordination nodes. Second, the three benzimidazole groups can freely twist around the quaternary carbon atom and the two –CH_2_– groups to match the requirements of various coordination geometries.

### Experimental

To a solution of 1*H*-benzimidazole (30 mmol) in 100 ml of dry THF/DMSO (10:1 *v*/*v*) under nitrogen was added sodium hydroxide (30 mmol) with vigorous stirring at 60 °C. After 1/2 h a solution of tetrakis(bromomethyl)methane(10 mmol) in 20 ml of THF was added over a period of 1 h. The reaction mixture was stirred continuously overnight at 80 °C. A 100 ml portion of water was added to give a precipitant that was recrystallized in EtOH. Then colorless crystals suitable for X-ray diffraction analysis were grown by recrystallization from a 9:1 ethanol:water mixture (yield: 86%).

### Refinement

H atoms were included in calclulated positions and refined in a riding-model approximation with C—H distances ranging from 0.93Å to 0.97Å and *U*_iso_(H) = 1.2 *U*_eq_(C). The structure contains large voids, but the disordered solvent molecules could not be located from difference Fourier maps. The PLATON/SQUEEZE procedure (Spek, 2009) was used to account for the electron density in this region.

### Crystal data

**Table:**

C_26_H_23_BrN_6_	*Z* = 2
*M**_r_* = 499.41	*F*(000) = 512
Triclinic, *P*1	*D*_x_ = 1.208 Mg m^−^^3^
Hall symbol: -P 1	Mo *K*α radiation, λ = 0.71073 Å
*a* = 9.297 (4) Å	Cell parameters from 2851 reflections
*b* = 11.869 (5) Å	θ = 2.2–22.5°
*c* = 13.661 (6) Å	µ = 1.52 mm^−^^1^
α = 68.956 (4)°	*T* = 293 K
β = 77.398 (4)°	Block, colourless
γ = 84.805 (4)°	0.32 × 0.30 × 0.29 mm
*V* = 1372.9 (10) Å^3^	

### Data collection

**Table:**

Bruker APEXII CCD diffractometer	5012 independent reflections
Radiation source: fine-focus sealed tube	3417 reflections with *I* > 2σ(*I*)
graphite	*R*_int_ = 0.025
φ and ω scans	θ_max_ = 25.5°, θ_min_ = 2.2°
Absorption correction: multi-scan (*SADABS*; Sheldrick, 2008*a*)	*h* = −10→11
*T*_min_ = 0.642, *T*_max_ = 0.667	*k* = −14→14
9233 measured reflections	*l* = −12→16

### Refinement

**Table:**

Refinement on *F*^2^	Secondary atom site location: difference Fourier map
Least-squares matrix: full	Hydrogen site location: inferred from neighbouring sites
*R*[*F*^2^ > 2σ(*F*^2^)] = 0.037	H-atom parameters constrained
*wR*(*F*^2^) = 0.086	*w* = 1/[σ^2^(*F*_o_^2^) + (0.040*P*)^2^] where *P* = (*F*_o_^2^ + 2*F*_c_^2^)/3
*S* = 1.01	(Δ/σ)_max_ < 0.001
5012 reflections	Δρ_max_ = 0.41 e Å^−^^3^
299 parameters	Δρ_min_ = −0.20 e Å^−^^3^
0 restraints	Extinction correction: *SHELXL97* (Sheldrick, 2008a), Fc^*^=kFc[1+0.001xFc^2^λ^3^/sin(2θ)]^-1/4^
Primary atom site location: structure-invariant direct methods	Extinction coefficient: 0.0027 (7)

### Special details

**Table:**

Geometry. All e.s.d.'s (except the e.s.d. in the dihedral angle between two l.s. planes) are estimated using the full covariance matrix. The cell e.s.d.'s are taken into account individually in the estimation of e.s.d.'s in distances, angles and torsion angles; correlations between e.s.d.'s in cell parameters are only used when they are defined by crystal symmetry. An approximate (isotropic) treatment of cell e.s.d.'s is used for estimating e.s.d.'s involving l.s. planes.
Refinement. Refinement of *F*^2^ against ALL reflections. The weighted *R*-factor *wR* and goodness of fit *S* are based on *F*^2^, conventional *R*-factors *R* are based on *F*, with *F* set to zero for negative *F*^2^. The threshold expression of *F*^2^ > σ(*F*^2^) is used only for calculating *R*-factors(gt) *etc*. and is not relevant to the choice of reflections for refinement. *R*-factors based on *F*^2^ are statistically about twice as large as those based on *F*, and *R*- factors based on ALL data will be even larger.

### Fractional atomic coordinates and isotropic or equivalent isotropic displacement parameters (Å^2^)

**Table:**

	*x*	*y*	*z*	*U*_iso_*/*U*_eq_	
Br1	0.02589 (3)	0.37659 (2)	0.17904 (2)	0.05951 (14)	
C18	0.1102 (3)	0.7601 (2)	0.0752 (2)	0.0474 (7)	
H18A	0.1910	0.8026	0.0803	0.057*	
H18B	0.1263	0.7613	0.0023	0.057*	
C9	0.1151 (3)	0.6271 (2)	0.15110 (19)	0.0398 (6)	
C26	0.0088 (3)	0.5573 (2)	0.1246 (2)	0.0475 (7)	
H26A	0.0211	0.5846	0.0473	0.057*	
H26B	−0.0907	0.5791	0.1523	0.057*	
C10	0.0700 (3)	0.6261 (2)	0.26770 (19)	0.0463 (6)	
H10A	0.1418	0.6722	0.2791	0.056*	
H10B	−0.0242	0.6680	0.2748	0.056*	
C8	0.2758 (3)	0.5812 (2)	0.13182 (18)	0.0430 (6)	
H8A	0.3360	0.6227	0.1576	0.052*	
H8B	0.2794	0.4958	0.1735	0.052*	
C2	0.5675 (3)	0.7424 (3)	−0.2327 (2)	0.0725 (10)	
H2	0.5874	0.7320	−0.2987	0.087*	
C5	0.5050 (3)	0.7772 (3)	−0.0330 (2)	0.0596 (8)	
H5	0.4843	0.7890	0.0324	0.072*	
C1	0.4692 (3)	0.6679 (3)	−0.1461 (2)	0.0540 (7)	
C6	0.4383 (3)	0.6874 (2)	−0.0490 (2)	0.0449 (6)	
N2	0.3398 (2)	0.59903 (18)	0.01862 (16)	0.0443 (5)	
C3	0.6335 (4)	0.8309 (4)	−0.2175 (3)	0.0846 (11)	
H3	0.7004	0.8812	−0.2739	0.101*	
N1	0.3930 (3)	0.5689 (2)	−0.1371 (2)	0.0638 (7)	
C7	0.3198 (3)	0.5320 (3)	−0.0377 (2)	0.0541 (7)	
H7	0.2592	0.4650	−0.0088	0.065*	
N5	−0.0269 (2)	0.82613 (18)	0.09621 (16)	0.0451 (5)	
C21	−0.0100 (4)	1.0452 (3)	0.2290 (3)	0.0700 (9)	
H21	0.0508	1.0786	0.2568	0.084*	
C19	−0.0459 (3)	0.9106 (2)	0.14554 (19)	0.0411 (6)	
C24	−0.1927 (3)	0.9483 (2)	0.1476 (2)	0.0473 (7)	
C20	0.0481 (3)	0.9582 (2)	0.1851 (2)	0.0574 (8)	
H20	0.1461	0.9332	0.1826	0.069*	
C22	−0.1552 (4)	1.0840 (2)	0.2327 (2)	0.0658 (8)	
H22	−0.1899	1.1435	0.2618	0.079*	
C25	−0.1577 (3)	0.8192 (2)	0.0705 (2)	0.0570 (7)	
H25	−0.1722	0.7694	0.0343	0.068*	
N6	−0.2618 (3)	0.8885 (2)	0.1011 (2)	0.0629 (7)	
C23	−0.2491 (3)	1.0351 (2)	0.1936 (2)	0.0609 (8)	
H23	−0.3476	1.0594	0.1977	0.073*	
N3	0.0574 (2)	0.50904 (18)	0.35181 (15)	0.0449 (5)	
C15	0.1751 (4)	0.2541 (3)	0.5510 (2)	0.0690 (9)	
H15	0.1316	0.1831	0.6008	0.083*	
C11	0.1656 (3)	0.4432 (2)	0.40562 (19)	0.0449 (6)	
C14	0.3172 (4)	0.2784 (3)	0.5444 (3)	0.0776 (10)	
H14	0.3712	0.2230	0.5908	0.093*	
N4	−0.0525 (3)	0.3426 (2)	0.47523 (18)	0.0602 (6)	
C17	−0.0684 (3)	0.4455 (3)	0.3981 (2)	0.0558 (7)	
H17	−0.1580	0.4720	0.3774	0.067*	
C16	0.0956 (3)	0.3389 (2)	0.4807 (2)	0.0524 (7)	
C13	0.3842 (4)	0.3829 (3)	0.4708 (3)	0.0735 (9)	
H13	0.4817	0.3964	0.4695	0.088*	
C12	0.3103 (3)	0.4681 (3)	0.3990 (2)	0.0558 (7)	
H12	0.3556	0.5381	0.3490	0.067*	
C4	0.6031 (3)	0.8479 (3)	−0.1189 (3)	0.0792 (10)	
H4	0.6508	0.9090	−0.1113	0.095*	

### Atomic displacement parameters (Å^2^)

**Table:**

	*U*^11^	*U*^22^	*U*^33^	*U*^12^	*U*^13^	*U*^23^
Br1	0.0688 (2)	0.05000 (18)	0.0600 (2)	−0.00572 (13)	−0.00812 (15)	−0.02107 (14)
C18	0.0433 (16)	0.0465 (14)	0.0429 (15)	0.0003 (12)	0.0019 (12)	−0.0106 (12)
C9	0.0348 (14)	0.0419 (13)	0.0385 (14)	−0.0003 (11)	0.0007 (11)	−0.0138 (12)
C26	0.0453 (15)	0.0486 (15)	0.0469 (16)	0.0027 (12)	−0.0081 (13)	−0.0160 (13)
C10	0.0509 (16)	0.0438 (14)	0.0379 (15)	0.0012 (12)	−0.0021 (12)	−0.0110 (12)
C8	0.0403 (15)	0.0491 (14)	0.0373 (15)	0.0027 (12)	−0.0040 (12)	−0.0151 (12)
C2	0.0505 (19)	0.103 (3)	0.0463 (19)	0.0172 (19)	−0.0022 (16)	−0.0139 (19)
C5	0.0447 (17)	0.076 (2)	0.0490 (18)	−0.0088 (15)	−0.0048 (14)	−0.0120 (16)
C1	0.0388 (16)	0.0732 (19)	0.0470 (18)	0.0132 (14)	−0.0046 (13)	−0.0229 (16)
C6	0.0312 (14)	0.0562 (16)	0.0407 (16)	0.0026 (12)	−0.0033 (12)	−0.0120 (13)
N2	0.0353 (12)	0.0538 (13)	0.0438 (13)	0.0039 (10)	−0.0035 (10)	−0.0208 (11)
C3	0.047 (2)	0.111 (3)	0.064 (2)	−0.0079 (19)	0.0009 (17)	0.002 (2)
N1	0.0553 (15)	0.0879 (19)	0.0571 (16)	0.0131 (14)	−0.0085 (13)	−0.0406 (15)
C7	0.0444 (17)	0.0694 (18)	0.0553 (19)	0.0089 (14)	−0.0068 (15)	−0.0341 (16)
N5	0.0389 (13)	0.0449 (12)	0.0477 (13)	0.0017 (10)	−0.0091 (10)	−0.0117 (11)
C21	0.077 (2)	0.0537 (18)	0.087 (2)	−0.0039 (17)	−0.0230 (19)	−0.0290 (18)
C19	0.0403 (15)	0.0350 (13)	0.0406 (15)	−0.0025 (11)	−0.0032 (12)	−0.0068 (12)
C24	0.0426 (16)	0.0414 (14)	0.0509 (16)	0.0054 (12)	−0.0074 (13)	−0.0101 (13)
C20	0.0459 (17)	0.0478 (16)	0.074 (2)	−0.0052 (13)	−0.0127 (15)	−0.0149 (15)
C22	0.079 (2)	0.0481 (17)	0.069 (2)	0.0018 (16)	−0.0088 (18)	−0.0223 (16)
C25	0.0565 (19)	0.0576 (17)	0.0657 (19)	0.0096 (15)	−0.0276 (16)	−0.0254 (15)
N6	0.0518 (15)	0.0614 (15)	0.0823 (18)	0.0124 (12)	−0.0266 (13)	−0.0287 (14)
C23	0.0544 (18)	0.0502 (16)	0.0648 (19)	0.0089 (14)	−0.0052 (15)	−0.0104 (15)
N3	0.0456 (13)	0.0468 (12)	0.0356 (12)	−0.0061 (10)	0.0002 (10)	−0.0100 (10)
C15	0.098 (3)	0.0493 (17)	0.0428 (18)	0.0025 (18)	−0.0012 (18)	−0.0049 (14)
C11	0.0558 (18)	0.0424 (14)	0.0349 (15)	−0.0002 (13)	−0.0059 (13)	−0.0135 (12)
C14	0.087 (3)	0.076 (2)	0.059 (2)	0.016 (2)	−0.018 (2)	−0.0117 (19)
N4	0.0685 (18)	0.0572 (15)	0.0433 (14)	−0.0157 (12)	0.0054 (12)	−0.0100 (12)
C17	0.0486 (17)	0.0661 (19)	0.0484 (17)	−0.0073 (14)	0.0019 (14)	−0.0198 (16)
C16	0.063 (2)	0.0474 (16)	0.0401 (16)	−0.0034 (14)	0.0019 (14)	−0.0144 (14)
C13	0.070 (2)	0.092 (2)	0.063 (2)	0.0081 (19)	−0.0207 (18)	−0.030 (2)
C12	0.0565 (19)	0.0617 (17)	0.0452 (17)	0.0004 (14)	−0.0085 (14)	−0.0150 (14)
C4	0.052 (2)	0.090 (2)	0.079 (3)	−0.0160 (17)	−0.0116 (18)	−0.008 (2)

### Geometric parameters (Å, °)

**Table:**

Br1—C26	2.005 (3)	N5—C19	1.375 (3)
C18—N5	1.463 (3)	C21—C22	1.383 (4)
C18—C9	1.550 (3)	C21—C20	1.384 (4)
C18—H18A	0.9700	C21—H21	0.9300
C18—H18B	0.9700	C19—C20	1.375 (4)
C9—C26	1.522 (3)	C19—C24	1.395 (3)
C9—C8	1.543 (3)	C24—N6	1.381 (3)
C9—C10	1.552 (3)	C24—C23	1.398 (4)
C26—H26A	0.9700	C20—H20	0.9300
C26—H26B	0.9700	C22—C23	1.379 (4)
C10—N3	1.445 (3)	C22—H22	0.9300
C10—H10A	0.9700	C25—N6	1.310 (3)
C10—H10B	0.9700	C25—H25	0.9300
C8—N2	1.475 (3)	C23—H23	0.9300
C8—H8A	0.9700	N3—C17	1.354 (3)
C8—H8B	0.9700	N3—C11	1.389 (3)
C2—C3	1.362 (5)	C15—C14	1.354 (4)
C2—C1	1.393 (4)	C15—C16	1.401 (4)
C2—H2	0.9300	C15—H15	0.9300
C5—C4	1.370 (4)	C11—C12	1.380 (4)
C5—C6	1.383 (4)	C11—C16	1.397 (3)
C5—H5	0.9300	C14—C13	1.382 (4)
C1—N1	1.381 (4)	C14—H14	0.9300
C1—C6	1.393 (4)	N4—C17	1.316 (3)
C6—N2	1.379 (3)	N4—C16	1.391 (4)
N2—C7	1.337 (3)	C17—H17	0.9300
C3—C4	1.397 (5)	C13—C12	1.383 (4)
C3—H3	0.9300	C13—H13	0.9300
N1—C7	1.313 (3)	C12—H12	0.9300
C7—H7	0.9300	C4—H4	0.9300
N5—C25	1.354 (3)		
			
N5—C18—C9	115.32 (19)	C25—N5—C18	128.1 (2)
N5—C18—H18A	108.4	C19—N5—C18	125.5 (2)
C9—C18—H18A	108.4	C22—C21—C20	122.2 (3)
N5—C18—H18B	108.4	C22—C21—H21	118.9
C9—C18—H18B	108.4	C20—C21—H21	118.9
H18A—C18—H18B	107.5	C20—C19—N5	132.8 (2)
C26—C9—C8	112.7 (2)	C20—C19—C24	122.2 (2)
C26—C9—C18	107.1 (2)	N5—C19—C24	105.0 (2)
C8—C9—C18	107.56 (18)	N6—C24—C19	110.6 (2)
C26—C9—C10	112.5 (2)	N6—C24—C23	129.6 (3)
C8—C9—C10	109.21 (19)	C19—C24—C23	119.8 (3)
C18—C9—C10	107.49 (19)	C19—C20—C21	116.9 (3)
C9—C26—Br1	117.62 (17)	C19—C20—H20	121.5
C9—C26—H26A	107.9	C21—C20—H20	121.5
Br1—C26—H26A	107.9	C23—C22—C21	120.5 (3)
C9—C26—H26B	107.9	C23—C22—H22	119.7
Br1—C26—H26B	107.9	C21—C22—H22	119.7
H26A—C26—H26B	107.2	N6—C25—N5	114.2 (3)
N3—C10—C9	116.5 (2)	N6—C25—H25	122.9
N3—C10—H10A	108.2	N5—C25—H25	122.9
C9—C10—H10A	108.2	C25—N6—C24	103.8 (2)
N3—C10—H10B	108.2	C22—C23—C24	118.4 (3)
C9—C10—H10B	108.2	C22—C23—H23	120.8
H10A—C10—H10B	107.3	C24—C23—H23	120.8
N2—C8—C9	113.94 (19)	C17—N3—C11	106.5 (2)
N2—C8—H8A	108.8	C17—N3—C10	125.3 (2)
C9—C8—H8A	108.8	C11—N3—C10	128.1 (2)
N2—C8—H8B	108.8	C14—C15—C16	118.0 (3)
C9—C8—H8B	108.8	C14—C15—H15	121.0
H8A—C8—H8B	107.7	C16—C15—H15	121.0
C3—C2—C1	117.7 (3)	C12—C11—N3	132.1 (2)
C3—C2—H2	121.1	C12—C11—C16	122.6 (2)
C1—C2—H2	121.1	N3—C11—C16	105.1 (2)
C4—C5—C6	116.3 (3)	C15—C14—C13	121.9 (3)
C4—C5—H5	121.8	C15—C14—H14	119.0
C6—C5—H5	121.8	C13—C14—H14	119.0
N1—C1—C6	109.9 (2)	C17—N4—C16	104.3 (2)
N1—C1—C2	130.2 (3)	N4—C17—N3	113.9 (3)
C6—C1—C2	120.0 (3)	N4—C17—H17	123.0
N2—C6—C5	132.1 (3)	N3—C17—H17	123.0
N2—C6—C1	105.3 (2)	N4—C16—C11	110.1 (2)
C5—C6—C1	122.5 (3)	N4—C16—C15	130.4 (3)
C7—N2—C6	106.4 (2)	C11—C16—C15	119.3 (3)
C7—N2—C8	127.9 (2)	C14—C13—C12	121.8 (3)
C6—N2—C8	125.7 (2)	C14—C13—H13	119.1
C2—C3—C4	121.5 (3)	C12—C13—H13	119.1
C2—C3—H3	119.3	C11—C12—C13	116.2 (3)
C4—C3—H3	119.3	C11—C12—H12	121.9
C7—N1—C1	104.1 (2)	C13—C12—H12	121.9
N1—C7—N2	114.3 (3)	C5—C4—C3	121.9 (3)
N1—C7—H7	122.9	C5—C4—H4	119.0
N2—C7—H7	122.9	C3—C4—H4	119.0
C25—N5—C19	106.4 (2)		
			
N5—C18—C9—C26	69.8 (3)	N5—C19—C24—N6	0.1 (3)
N5—C18—C9—C8	−168.8 (2)	C20—C19—C24—C23	−1.6 (4)
N5—C18—C9—C10	−51.3 (3)	N5—C19—C24—C23	179.8 (2)
C8—C9—C26—Br1	46.0 (3)	N5—C19—C20—C21	178.9 (3)
C18—C9—C26—Br1	164.16 (16)	C24—C19—C20—C21	0.8 (4)
C10—C9—C26—Br1	−77.9 (2)	C22—C21—C20—C19	−0.4 (4)
C26—C9—C10—N3	58.1 (3)	C20—C21—C22—C23	1.0 (5)
C8—C9—C10—N3	−67.7 (3)	C19—N5—C25—N6	−1.9 (3)
C18—C9—C10—N3	175.8 (2)	C18—N5—C25—N6	179.1 (2)
C26—C9—C8—N2	64.9 (3)	N5—C25—N6—C24	1.9 (3)
C18—C9—C8—N2	−53.0 (3)	C19—C24—N6—C25	−1.2 (3)
C10—C9—C8—N2	−169.4 (2)	C23—C24—N6—C25	179.2 (3)
C3—C2—C1—N1	−176.6 (3)	C21—C22—C23—C24	−1.8 (4)
C3—C2—C1—C6	1.8 (4)	N6—C24—C23—C22	−178.3 (3)
C4—C5—C6—N2	177.7 (3)	C19—C24—C23—C22	2.1 (4)
C4—C5—C6—C1	0.7 (4)	C9—C10—N3—C17	−93.6 (3)
N1—C1—C6—N2	−0.9 (3)	C9—C10—N3—C11	90.9 (3)
C2—C1—C6—N2	−179.5 (2)	C17—N3—C11—C12	−174.1 (3)
N1—C1—C6—C5	176.8 (2)	C10—N3—C11—C12	2.1 (4)
C2—C1—C6—C5	−1.8 (4)	C17—N3—C11—C16	2.1 (3)
C5—C6—N2—C7	−176.0 (3)	C10—N3—C11—C16	178.3 (2)
C1—C6—N2—C7	1.4 (3)	C16—C15—C14—C13	−0.3 (5)
C5—C6—N2—C8	1.7 (4)	C16—N4—C17—N3	0.6 (3)
C1—C6—N2—C8	179.1 (2)	C11—N3—C17—N4	−1.7 (3)
C9—C8—N2—C7	−79.6 (3)	C10—N3—C17—N4	−178.1 (2)
C9—C8—N2—C6	103.2 (3)	C17—N4—C16—C11	0.8 (3)
C1—C2—C3—C4	−0.7 (5)	C17—N4—C16—C15	176.5 (3)
C6—C1—N1—C7	0.1 (3)	C12—C11—C16—N4	174.8 (2)
C2—C1—N1—C7	178.5 (3)	N3—C11—C16—N4	−1.8 (3)
C1—N1—C7—N2	0.9 (3)	C12—C11—C16—C15	−1.5 (4)
C6—N2—C7—N1	−1.5 (3)	N3—C11—C16—C15	−178.1 (2)
C8—N2—C7—N1	−179.1 (2)	C14—C15—C16—N4	−174.1 (3)
C9—C18—N5—C25	−77.3 (3)	C14—C15—C16—C11	1.3 (4)
C9—C18—N5—C19	103.9 (3)	C15—C14—C13—C12	−0.7 (5)
C25—N5—C19—C20	−177.4 (3)	N3—C11—C12—C13	176.1 (3)
C18—N5—C19—C20	1.6 (4)	C16—C11—C12—C13	0.5 (4)
C25—N5—C19—C24	1.0 (3)	C14—C13—C12—C11	0.6 (5)
C18—N5—C19—C24	180.0 (2)	C6—C5—C4—C3	0.4 (4)
C20—C19—C24—N6	178.7 (2)	C2—C3—C4—C5	−0.4 (5)

## References

[bb1] Barnett, S. A. & Champness, N. R. (2003). *Coord. Chem. Rev.* **246**, 145–168.

[bb2] Bradshaw, D., Claridge, J. B., Cussen, E. J., Prior, T. J. & Rosseinsky, M. J. (2005). *Acc. Chem. Res.* **38**, 273–282. 10.1021/ar0401606 15835874

[bb3] Bruker (2008). *SAINT* and *APEX2* Bruker AXS Inc., Madison, Wisconsin, USA.

[bb13] Clegg, W. & Martin, N. C. (2007). *Acta Cryst* E**63**, m856.

[bb4] Ferey, G., Mellot-Draznieks, C., Serre, C. & Millange, F. (2005). *Acc. Chem. Res.* **38**, 217–225. 10.1021/ar040163i 15835868

[bb5] Ko, J. W., Min, K. S. & Suh, M. P. (2002). *Inorg. Chem.* **41**, 2151–2157. 10.1021/ic011281u 11952368

[bb6] Liu, H. Y., Wu, H., Ma, J. F., Liu, Y. Y., Liu, B. & Yang, J. (2010). *Cryst. Growth Des.* **10**, 4795–4805.

[bb7] Pan, L., Olson, D. H., Ciemnolonski, L. R., Heddy, R. & Li, J. (2006). *Angew. Chem. Int. Ed.* **45**, 616–619. 10.1002/anie.200503503 16355421

[bb8] Pan, L., Sander, M. B., Huang, X., Li, J., Smith, M. R. Jr, Bittner, E. W., Bockrath, B. C. & Johnson, J. K. (2004). *J. Am. Chem. Soc.* **126**, 1308–1309. 10.1021/ja0392871 14759166

[bb9] Sheldrick, G. M. (2008*a*). *SADABS* University of Göttingen, Germany.

[bb10] Sheldrick, G. M. (2008*b*). *Acta Cryst.* A**64**, 112–122. 10.1107/S0108767307043930 18156677

[bb11] Spek, A. L. (2009). *Acta Cryst.* D**65**, 148–155. 10.1107/S090744490804362X PMC2631630 19171970

[bb12] Yang, X. P., Kang, B. S., Wong, W. K., Su, C. Y. & Liu, H. Q. (2003). *Inorg. Chem.* **42**, 169–179. 10.1021/ic025799p 12513092

